# Driver genes in non-small cell lung cancer: Characteristics, detection methods, and targeted therapies

**DOI:** 10.18632/oncotarget.17016

**Published:** 2017-04-10

**Authors:** Qing-Ge Zhu, Shi-Ming Zhang, Xiao-Xiao Ding, Bing He, Hu-Qin Zhang

**Affiliations:** ^1^ The Key Laboratory of Biomedical Information Engineering of Ministry of Education, School of Life Science and Technology, Xi’an Jiaotong University, Xi’an 710049, P.R. China

**Keywords:** driver genes, non-small cell lung cancer, characteristics, detection methods, targeted therapies

## Abstract

Lung cancer is one of the most common causes of cancer-related death in the world. The large number of lung cancer cases is non-small cell lung cancer (NSCLC), which approximately accounting for 75% of lung cancer. Over the past years, our comprehensive knowledge about the molecular biology of NSCLC has been rapidly enriching, which has promoted the discovery of driver genes in NSCLC and directed FDA-approved targeted therapies. Of course, the targeted therapies based on driver genes provide a more exact option for advanced non-small cell lung cancer, improving the survival rate of patients. Now, we will review the landscape of driver genes in NSCLC including the characteristics, detection methods, the application of target therapy and challenges.

## INTRODUCTION

By far, the number one with the increasingly rapid incidence rate worldwide among all tumors is lung cancer, which has the highest morbidity rate. The incidence of lung cancer primarily results from long-term tobacco smoking (85%) [1–6], while there are about 10%–15% of patients who have never smoked [7], in that non-smokers may be exposed to second-hand smoke, radon gas, asbestos, air pollution, and so on. Certainly, about 8% of lung cancer is the consequence of genetic factors [8] that enhance the risk of disease occurring. In accordance with histological type, lung cancer can be departed into two main subtypes, small cell lung cancer (SCLC) and non-small cell lung cancer (NSCLC).

With the discovery of driver genes, non-small cell lung cancer is subdivided including adenocarcinoma, squamous cell carcinoma, large cell carcinoma, and the other unspecified. Over the past decade, it has proved that the classification contributed to choose the optimal therapy.

In general case, about 41% of patients who are attacked by this disease are confirmed during the IV stage of NSCLC (Table 1). Chemotherapy was previously the conclusive recommendations, but it had the low cure rate and brought patients bad side effects and miserable experiences. Worst yet, compared to small cell carcinoma, NSCLC relatively lacks sensitivity to chemotherapy. Now, with the accumulation of our knowledge about driver genes, targeted therapies against driver genes have provided a better choice for advanced patients, which has much better treatment effects and lower side effects. As we all known, there are only an overall 5-year survival of 15% [9] for all stages. Thus, it is very urgent to improve the early diagnosis and effective treatments to increase the survival rate of patients. Under the circumstances, the study about driver genes has been a crucial breakthrough to solve these current problems.

**Table 1:** Current treatment recommendation of NSCLCs

Stage*	General treatment recommendations
IA	Surgical resection
IB	Surgical resection, can consider adjuvant chemotherapy in selected cases (e.g. tumor size > 4 cm)
IIA	Surgical resection followed by adjuvant chemotherapy
IIB	Surgical resection followed by adjuvant chemotherapy
IIIA	Multimodality treatment: chemotherapy, radiation, +/− surgery
IIIB	Multimodality treatment: chemotherapy and radiation
IV	Chemotherapy, consider targeted therapies according to driver mutations

*described according to TNM.

Following the appearance of Next-generation sequencing technologies, many genes show the characters that can influence the growth, survival, and migration of tumors.

Further research into Cancer Genomic Projects has discovered that genetic abnormalities named driver gene mutation, including gene mutations and gene rearrangements, were directly or indirectly implicated in oncogenesis [10]. Mut-driver gene contains driver gene mutations. Recently, some researchers defined the gene expressed aberrantly and behaving a selective growth advantage in cancers as Epi-driver gene. Of course, both driver gene mutation and Epi-driver gene were driver genes. These driver genes may affect the signaling pathways regulating the core cellular processes (cell fate, cell survival, genome maintenance). In order to enhance the curative ratio of NSCLC, we need to clearly know more driver genes about the relationship between each other, how to influence NSCLC, and targeted therapies.

### How to identify driver genes?

As we all known, identifying driver genes in a typical tumor is critical to promote the development about clinical therapeutics. Now, there have been two databases to identify driver gene, Driver DB [11] (an exome sequencing database for cancer driver gene identification) and the Candidate Cancer Gene Database [12] (a database of cancer driver gene from forward genetic screens in mice), because of the advance of exome sequencing [13, 14]. Driver DB provides the calculated results about screening driver genes for a cancer by eight algorithms, the explanation on relationships among driver genes (Gene Oncology, Pathway and Protein/Genetics Interaction), the different mutation information of per driver gene, Meta-Analysis function, and so on. While the Candidate Cancer Gene Databases (CCGD) includes a unified description of candidate driver genes overall recently published and the genomic locations, which are transposon common insertion sites originated from transposon-based screens. The arising of these databases have not only brought the great convenience for the identification about driver genes but also furthered the efficiency of cancer research.

### The prediction of driver genes

Accompanying with the arising of NGS and extensive data sets derived from cancer omics, there have been diversified methods to predict driver genes in a special tumor, profiling [15]. At present, two major strategies based on the mutation frequency or functional analysis of variant protein originated from gene mutation apply to identify and predict driver genes. Generally, the former infers whether one gene is driver gene by the means of comparing the mutation frequency of single locus or other loci between the same or similar cancer [16, 17]. Whereas some researchers think that this method on the strength of the pattern of mutation is superior to the one based on mutation frequency. Extremely characteristic, as well as nonrandom, is the patterns of mutation about suppressor genes and oncogenes, which was well studied. Thus, the patterns of mutation can make us rapid to classify one driver gene as oncogene or suppressor gene, contributing to the next step research. However, how to distinguish oncogenes from suppressor gene just according to the pattern of mutation remains to be further studied [18].

While the latter predict driver genes through inferring the function of variant protein generated from genes mutation [19]. It is easy for the mut-driver genes with high mutation frequency to identify through the method based on mutation frequency, yet, which is not suitable for the mut-driver genes that possess low frequency and play a crucial role in the tumor genesis. This problem is overcame by the method based on the function analysis of variant protein. In fact, it is impossible for all variant protein to confer the selective growth advantage, which is the severe weakness of the functional analysis. Similarly, there are a large of mutation genes with high mutation frequency but helpless to the development of tumor. In conclusion, the driver genes predicted by these strategies remains subsequent analysis and experimental verification.

As for Epi-driver gene, the further analysis of differential expressed genes through comparing the expression of genes between cancer tissues and normal tissues of change is the dominating strategy to identify the driver gene. The mutation of epi-driver genes often occurred during the proliferation of cells because it is the phase that DNA or chromatin is prone to be damaged by DNA methylation, histone modification (histone methylation and histone acetylation), and the DNA repair dysregulation [29]. In addition, the genes expression may be interrelated with ages of organisms, cell type, and environmental factors besides regulatory factors. Therefore, how to distinguish epi-driver genes from other factors who result in the variation of genes expression is a significant challenge to identify driver genes.

Surely, there have been emerging many algorithms to screen driver gene. For example, Lei Chen et proposed a computational method to identify lung adenocarcinoma drivers according to the methylation, mutation, microRNA, and mRNA levels on the dysfunctional genes [20]. However, none of the existing algorithms, at present, became the gold standard. Every algorism has own too special stresses and weakness to easily make comparisons about the results predicting driver genes by different algorithms; that is, it is the best choice for these algorisms to be used to screen driver genes in order to further analysis but not identification. Now, some reviews and databases display the outcomes of several algorithms and even form a system that have a relatively higher accuracy on the predicting driver genes for a specific cancer. DriverDBv2 have published bioinformatics algorithms dedicated to driver gene or mutation identification; the ‘Cancer’ section summarizes the calculated results about driver genes by 15 computational methods for a specific cancer type or dataset and even provides three levels of biological interpretation for realization of the relationships between driver genes. Collin J. Tokeheim *et al*. compared eight algorithms regarding overlap of the driver genes predicted by each method, the discrepancy between the expected p-values and the observed one, the number and consistencies predicting driver genes, variability respectively in background mutation number and in radiometric features, and evaluating the evaluation of cancer driver genes. Although these efforts have promoted the prediction of driver genes, the accuracy remains to be increased.

### The detection of driver genes

Besides the above methods, experimental methods can detect and conform driver genes, and have the more far-reaching significant. For example, EGFR mutation and ALK gene rearrangement, the driver genes of pulmonary adenocarcinomas, have been well tested and verified by experimental methods [21, 22]. On genome, most laboratories usually believe fluorescence *in situ* hybridization (FISH) assays. Originally, the detection of ROS1 gene rearrangement is by the mean of dual break-part fluorescence *in situ* hybridization (FISH) probes. Recently non-*in situ* testing approaches, including next-generation sequencing (NGS) and real-time PCR assays, have been much potential to be independent methods or supplementary tests to detect driver genes. As for transcription, the course about mRNA resulting from the rearrangement gene, RTPCR have been the another approach. It is known that the ROS1 rearrangements only account for 1–2% of occurrence in NSCLC [19], which decreased the accuracy of laboratory findings. However, immunohistochemistry can solve the dilemma by detecting the increased ROS1 protein levels, providing a supplement for FISH screening test.

### Fluorescence *in situ* hybridization

Fluorescence *in situ* hybridization (FISH), rooting in radioactivity *in situ* hybridization technique during the late 1980s, is a nonradioactive molecular and cellular genetic technique with fluorescent tags instead of isotope labeling. From December 2007 to April 2011, fluorescence in-situ hybridization (FISH) have been screening EGFR gene variation from tumor samples derived from 149 subjects. And FISH test were successfully treated in the majority of 49 patients who conferred positive EGFR [23]. Thus, FISH play an unshakable role in detection and screening of driver genes. In general, the abnormalities about EGFR, ALK, RET and ROS1 are well validated by FISH because the results were widely accepted. The important portion in FISH is the design about dual-colour break-apart probes, which have the completely different fluorochromes in the 3′end and the 5′ end. In order to distinguish two genes such as ROS1(green fluorochrome at 3′end) and ALK (orange luorochrome at 3′) when they are run together on the same side, the selection about the fluorochrome color at 3′ end need to be cautious [24]. Otherwise, some reports indicate that the experimental material older than 6 months may induce the low binding efficiency of probes [25].

### Immunohistochemistry

Immunohistochemistry (IHC) is the course detecting special antigens (e.g. proteins) in some cells of a tissue section according to the rationale that antibodies specifically bind to proteins in biological tissues. Its name originates from the roots “immuno”, antibodies participating in the method, and “histo” meaning tissue. Albert Coons is the first person to conceptualize IHC and apply to practical laboratory in 1941.

If the level of detected genes is low, IHC with relatively sensitivity, depending on how to define the threshold, is a much effective screening tool. However, scoring IHC results have been not reaching a consensus due to the different outcomes from different scoring approaches, which is perfectly similar to FISH one [25]. In NSCLC, Immunohistochemistry often acts as a supplemented trial for the reason that IHC cannot quickly meanwhile test multiple samples and have higher cost than FISH.

### Non-*in situ* technologies

Compared with these experimental methods above, non-*in situ* technologies have been, to some extent, more meaningful and convenient. For now, there are RTPCR and NGS applied to detect driver genes in NSCLC [26–29].

Making easily, meanwhile processing multiple samples, relatively low-cost, and having high sensitivity even reaching 100% are the reason why most laboratories adopted RTPCR. To get the perfect outcome, we need to prudently design primers (a key factor to PCR) and assure easily depredated and polluted RNA quality. Although RTPCR, as with FISH, easily leaves out low expressed genes, a new method according to RTPCR turns up and has been used for detecting ALK gene by testing 3′ region about transcripts [30, 31]. In addition, a single-tube multiplexed method, namely a combination of fusion-specific and 3′overexpression detection strategy, have detected ROS1, RET, and ALK fusions in lung cancer [32]. RTPCR may be a new tendency applied to the study about driver genes in NSCLC.

Of course, NGS also have enormous potential for detection of driver genes and there are various new strategies on the strength of NGS [12]. Anchored multiplex PCR, adapted from next-generation sequencing, can effectively detect single nucleotide variants, gene rearrangements, deletions, insertions, and copy number changes. Compared to the reference methods, anchored multiplex PCR possesses 100% specificity and sensitivity and even have identified TRIM4-BRAF, SN-ROS1, TPM3-NTRK1 VAMP2-NRG1, and RUFY2-RET in lung cancer [33–35]. At the same time, hybrid capture-based NGS [36] identifying genomic alterations in lung adenocarcinomas have been used as a potent discovery tool and sketchy clinical assay in NSCLC. Similar to RTPCR, NGS can be a major strategy applied to the study about driver genes in NSCLC.

In summary, whether the prediction or the detection of driver genes, there is no exclusive gold strategy generally acknowledged as only method to screen driver genes in NSCLC. As every strategy has weaknesses, perfecting a system (e.g. DriverDB) with several strategies developed to evaluate driver genes has been becoming a tendency. The research for strategies screening driver genes may accelerate later study about cancers and clinical detection.

### Driver genes in non-small cell lung cancer

About 75% of all lung cancers is NSCLC that have roughly 70 years on the median ages of the diagnosis [37–39]. What’s worse, more than half of lung cancers were diagnosed during advanced stages, an extremely mortal stages for patients who were often advised to receive chemotherapy and radiation, which possessed the low cure rate and very serious side effects [40–43]. Now, the research about driver genes have brought light to the complexion. Here, we briefly depict several typical driver genes in non-small lung cancer.

### Receptor tryosine kinases (RTKs)

**Figure 1:**
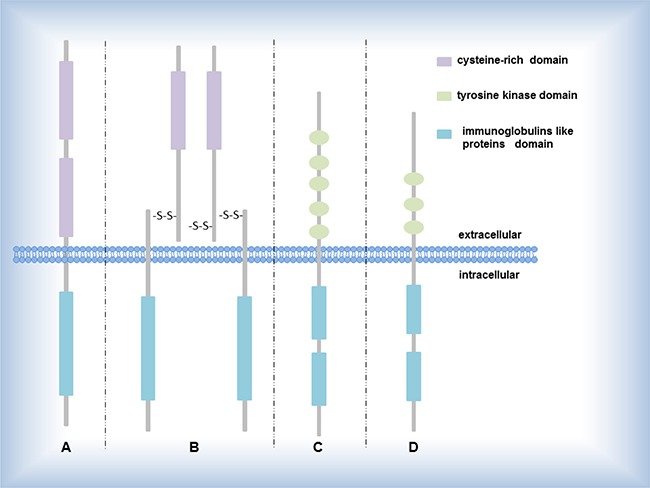
Several RTK subgroups (**A**) EGF receptor EGF, HER2 encode the proteins belonging to EGF receptor; (**B**) IGF-1 receptor ALK, ROS1 encode the proteins belonging to IGF-1 receptor; (**C**) PDGF receptor PDGFRA encode the proteins belonging to PDGF receptor; (**D**) FGF receptor FGF encodes the protein belonging to FGF receptor.

**Figure 2:**
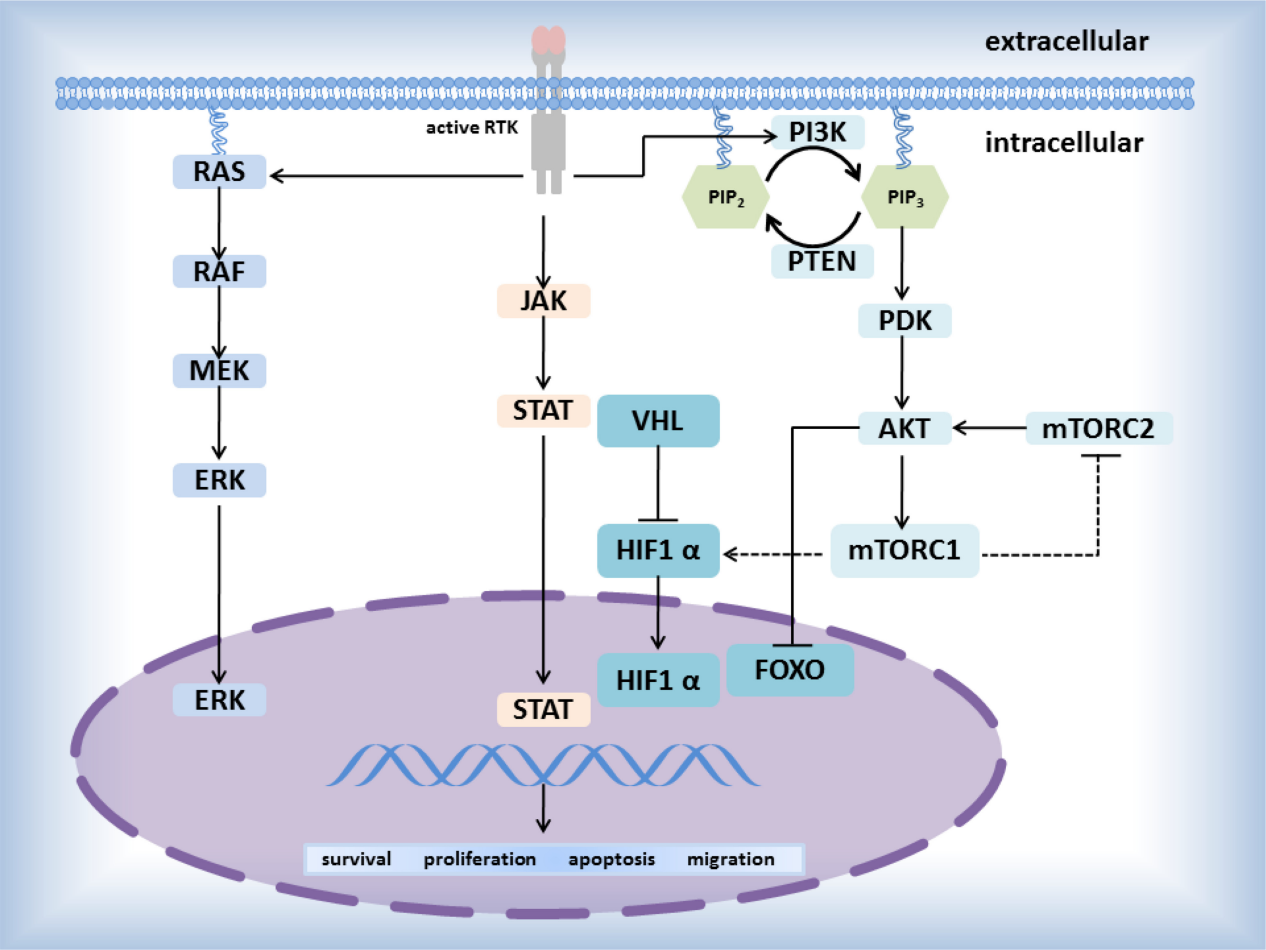
Pathways mediated by RTKs

Catalytic receptors, especially receptor tyrosine kinases (RTKs), playing a key figure in the mutual communication between cells and their microenvironments, affect and regulate metabolism and proliferation of cells by the special signaling pathways including RTK/Ras/MAPK signaling pathway, PI3K-PKB(Akt)signaling pathway-β-Smad signaling pathway, AK- STAT signaling pathway (Figure 2). Until now, several driver genes (Table 2) closely relate to transmembrane proteins belonging to RTKs (Figure 1), participate in the signaling pathway mediated by RTKs, and have been well-study as therapeutic targets in NSCLC [44].

**Table 2:** The brief description of several driver genes related to transmembrane proteins (RTKs)

EGFR	epidermal growth factor receptor; binding of the protein to a ligand induces receptor dimerization and tyrosine autophosphorylation and leads to cell proliferation. The most frequent mutations are a missense mutation at codon 858 (L858R) and in-frame deletions in exon 19 [46, 47, 49].
HER2	erb-b2 receptor tyrosine kinase 2; This gene encodes a member of the epidermal growth factor (EGF) receptor family of receptor tyrosine kinases. This protein has no ligand binding domain of its own and therefore cannot bind growth factors [51].
DDR2	discoidin domain receptor tyrosine kinase 2; RTKs have a tripartite structure with extracellular, transmembrane, and cytoplasmic regions, while this gene encodes a member of a novel subclass of RTKs and contains a distinct extracellular region encompassing a factor VIII-like domain [55].
ALK	anaplastic lymphoma receptor tyrosine kinase; belonging to the insulin receptor superfamily; This protein includes an extracellular domain, a hydrophobic stretch corresponding to a single pass transmembrane region, and an intracellular kinase domain. A typical example: a fusion gene combining EML4 and ALK [31].
ROS1	ROS proto-oncogene 1, receptor tyrosine kinase; belonging to the sevenless subfamily of tyrosine kinase insulin receptor genes; The protein encoded by this gene is a type I integral membrane protein with tyrosine kinase activity; The protein may function as a growth or differentiation factor receptor [32, 58, 84].
RET	ret proto-oncogene, a member of the cadherin superfamily, encodes one of the receptor tyrosine kinases [58, 84].
MET	MET proto-oncogene, receptor tyrosine kinase; This gene encodes a member of the receptor tyrosine kinase family of proteins and the product of the proto-oncogene MET [44].
FGFR1	fibroblast growth factor receptor 1; belonging to the fibroblast growth factor receptor (FGFR) family; the full-length representative protein consists of an extracellular region, composed of three immunoglobulin-like domains, a single hydrophobic membrane-spanning segment and a cytoplasmic tyrosine kinase domain [50, 59].
PDGFRA	platelet derived growth factor receptor alpha; This gene encodes a cell surface tyrosine kinase receptor for members of the platelet-derived growth factor family. The identity of the growth factor bound to a receptor monomer determines whether the functional receptor is a homodimer or a heterodimer, composed of both platelet-derived growth factor receptor alpha and beta polypeptides [50].

### EGFR, HER2, DDR2 (mutation)

EGFR [45–49] mutations located in the tyrosine kinase domain (Figure 1) may induce elementary EGFR signaling such as the RAS-MEK-ERK and PI3K-AKT pathways (Figure 2) that are crucial to the proliferation and migration of tumors. The discovery about activating EGFR mutations has unlocked a new stage during the evolution of the effective treatments for patients with NSCLC [46, 50–52]. With the appearance of the resistance against targeted EGFR therapy, current researches about EGFR principally focus on TKIs resistance and seeking the best way to overcome the resistance mechanisms, incorporating the EGFR T790M mutations (approximately 50% in resistant cancers), PIK3CA mutations, and MET amplification. So far, patients at the IV stage in NSCLC are determined whether receive EGFR TKIs depending on screening genotype of EGFR mutation. As with EGFR, the HER2 protein (also named ERBB-2) is a member of the HER family of receptor tyrosine kinases, which are easy to form homodimers with itself or heterodimers with the others of HER family [53, 54]. In addition, 20% in NSCLC is overexpressed the HER2 protein [46, 50–52]. The mutations generally involve inframe insertions mostly in exon 20 (e.g. mainly involved Tyr-Val-Met-Ala at codon 776), inducing the receptor constitutively active. In most cases, the HER2-positive is inhibited by small molecule agents targeted both the EGFR-positive and the HER2-positive (e.g. lapatinib) but not alone EGFR [46].

DDR2 (discoidin domain receptor tyrosine kinase 2) codes one of members of a novel subclass of RTKs (Figure 1) that may bind collagen and contribute to cell proliferation, migration, and survival. Like EGFR and HER2, the variation type of DDR2 is mutation and identified in SCC. Now DDR2 may be recognized as an oncogenic driver gene and susceptible to DDR2 kinase inhibitors [55].

### ALK, ROS1, RET (gene rearrangement)

In 2007 Soda M etc. [45] firstly discovered the EML4-ALK fusion gene from a 62-year-old patient with lung adenocarcinoma and confirmed EML4-ALK carcinogenicity in mice. It has been shown that the ALK- EML4 could be formed by more than 10 modes, the most prominent one of which is the fuse about EML4 gene exon 13 and ALK gene exon 20 [30–32, 56]. Similar to ALK rearrangements, the ROS1 rearrangements accounting for 1–2% in NSCLC could come up by ROS1 fusing with SDC4 (the other prominent partners: FIG, SLC34A2, CD74, and so on). Next, by the reason that ROSI owns reaching 49% amino acid sequence homology as with as ALK in the kinase domains [57], the clinical research about ROS1 often draws lessons from the one of ALK [5, 24, 25, 32, 57, 58]. For instance, Crizotinib, a tyrosine kinase inhibitor of ALK, is applied equally to the therapy for ROS1-positive patients with advanced NSCLC [5], which is approved by the FDA. Moreover, ROS1-positive, ALK-positive, and RET-positive cases are more arising in the never-smoker, younger, or patients with adenocarcinoma. Similar to ALK and ROS1, genetic alterations in RET involved chromosomal rearrangements that induced oncogenic transformation [32, 58]. At present, only targeted RET-positive inhibitors have been not yet developed, but drugs with multiple targets such as vandetanib, sorafenib have entered clinical trials as inhibitors. All of ALK, ROS1, and RET can involve in the signal pathway, including JAK-STAT3, RAS/MEK/ERK, PI3K/AKT, to regulate cellular survival and proliferation in NSCLC. In view of the similarities and differences among these genes, the steps to overcome cancers will be speeded.

### MET, FGFR1, PDGFRA (gene amplification)

First, the proteins encoded by these genes belong to enzyme-linked receptors (Figure 1) such as hepatocyte growth factor receptor (HGFR) (encoded by MET), fibroblast growth factor receptor 1(encoded by FGFR1) belonging to FGFR family, and platelet derived growth factor receptor alpha (encoded by PDGFRA). Second, the genes due to amplification may sustaining activating the downstream of some signaling pathways. Except these similar points, it is not surprise that everything has the unique side. By experimental methods, we can know that MET amplification, associated with the poor prognosis, occur in 2–4% of NSCLC. And 20% of EGFR-positive patients who show TKls indicate MET-positive [7]. FGFR1 amplification connected with smoking occurs in 15%–20% of lung SCC. And FGFR1 inhibitors is under development [59]. A research on the TKI “BGJ398” [49] with positive FGFR1 amplification among lung SCC patients displayed sectional response (15% of patients). As for PDGFRA, its amplification as a driver oncogene is easy to find in lung squamous cell cancers. Besides small molecule inhibitors, we can inhibit PDGFRA via the knockdown technology of shRNA. Like the FGFR1 inhibitors, multiple PDGFR agents cannot inhibit one kinase but multiple kinases. In order to be applied to the clinical, there still remains more efforts and further study [9]. In brief, these genes have many similarities such as proteins encoded by them and variation type, suggesting that these genes can mutually imitate each other when understand the molecule mechanism inducing cancers and seek the tactics to overcome cancers, which contributes to expedite the tenor of the NSCLC.

### Other components of signal pathways mediated by rtks

Besides the above driver genes about RTKs, the other components of signal pathways mediated by RTKs is often the targets. Now, we give a brief introduction about them (e.g. KRAS, BRAF, PIK3CA, PTEN).

### KRAS, BRAF (the signaling pathway of RAS/RAF/ERK)

KRAS (belonging to ras gene family) encodes the protein attached to the member of the GTPase superfamily. KRAS mutation occurring among∼25% of adenocarcinomas and localizing primarily in codons 12 and 13 is one of the most repeatedly mutated genes in NSCLC [60, 61]. KRAS mutations is non-overlapping with ALK rearrangement and EGFR mutations. KRAS mutations are associated with the signaling pathway of RAS/RAF/ERK [62–64]. Although there are, at present, no agents directly targeting KRAS, the strategies about combining PI3K or MEK inhibition and chemotherapy are ongoing.

BRAF (a proto-oncogene) encoding a Ser-Thr kinase protein which is a downstream effector protein in the RAS/RAF/ERK signaling pathway can directly phosphorylate the MEK and even activate the ERK to affect the cells proliferation and survival [65–69]. BRAF is one of members of the RAF kinase family including ARAF, BRAF, and RAF1 (also named cRAF) [70]. About 80% of BRAF mutations burst in exon 15 (the Val600 residue within the kinase domain), similar to the phosphorylation about T598 and S601 loci, continuingly activating BRAF protein [48]. It proves that BRAF mutation is mutually particular to KRAS and EGFR mutations. By far, the most promising small molecule inhibitor is for BRAF PLX4032 (Plexxikon, Berkeley, CA, USA). The other inhibitors are now under development.

### PIK3CA, PTEN (the signaling pathway of PI3K/Akt)

PIK3CA encodes the p110α isoform, the catalytic subunit of PI3K proteins [71] ; while PI3K proteins (lipid kinases) can regenerate phosphatidylinositol-3-phosphate mediating the downstream of PI3K/Akt pathway [72–74]. Mutational hotspots of PIK3CA generally cluster in exons 9 and 20(respectively encoding the helical and kinase domains of the protein). Multiple PI3K inhibitors have been under development. The hypothesis that PIK3CA mutation is susceptible to single-agent PI3K signaling pathway inhibitors in lung cancer remains to investigate.

As with PI3K, we know that phosphorylated mTOR actives the downstream of PI3K/Akt. However, PTEN (a suppressor gene) encoding a lipid phosphatase could negatively regulate PI3K/Akt signal pathway by inhibiting the activity of mTOR [9, 75, 76]. Thus, similar to the function of PI3K inhibitors, the loss of PTEN could activate PI3K/Akt signal pathway [77, 78]. PTEN is a potential target to develop a new therapy for NSCLC.

### Other driver genes

Besides driver genes above, there are some epi-driver genes possessing low frequency mutation but affecting the states of chromatin or DNA. For example, overexpression of H3F3A (encoding histone H3.3) is closely related with progression and migration of lung cancers via influencing metastasis-related genes [79, 80]. H3.3 could bind to the particular intronic region (e.g. GPR87) to modify the chromatin status and even directly activate special genes transcription (e.g. GPR87).H3.3 overexpression enhance the GPR87 IRE enhancer activity to promote the GPR87 transcription. GPR87 may be a feasible target gene for treatment because the encoding protein as G-protein-coupled receptor effectively plays important role in activing the downstream signaling pathway [81, 82]. At the same time, H3F3A may be a rough prognostic marker for early-stage lung cancer, which has the potential to form a therapy about GPR87 antagonists [80].

### The targeted therapies of non-samll cell lung cancer

Chemotherapy has been prior well approved as the sole strategy for all cancers but it has also brought some miserable side effects for patients because of compromising immune systems. The therapies of advanced NSCLC have been rapidly evolving in past years. With the discovery of driver genes, the new methods to classify NSCLC have been offered and there are more and more targets to cure NSCLC [79, 80]. Currently the list of driver genes remains to be increasing. The development of anti-EGFR, anti-VEGF, and anti-ALK therapies has been trialed and proven in NSCLC; the therapies targeted ROS1, MET, and BRAF are under study and own enormously potential to cure NSCLC; there are no currently effective therapies for these targets including KRAS, FGFR1, DDR2, and so on [83]. Even more recently, immuno-oncological therapies are the new ways for NSCLC according to the discovery of potential targets such as programmed death-1 receptor (PD-1) and cytotoxic T lymphocyte antigen-4 (CTLA-4) [83–85]. CTLA-4 signaling could barrier the start response of T cell in lymphnodes, while PD-1 expression could limit the activity of T cell in the microenvironment filled with cancer cells. At present, personalized medicine that patients choose their therapeutic schemes based on their special histological subtypes and molecular information gradually gets into the sight of people [12]. It is obvious standing to reason that the research about driver genes in NSCLC is quite essential for curing cancers. How to choose therapeutic regimen should depend on the conclusion of the diagnosis. The methods of clinical diagnosis are under the updating and some driver genes (Table 3) in NSCLC may be prognostic and predictive.

**Table 3:** The depiction of several driver genes

Gene	Location	Type of Alteration	Frequency	Function	Implication
EGFR	7p11.2	mutation	10–35%	activate the PI3K-AKT and RAS-MEK-ERK pathways central to the growth, survival, and migration of cancer cells.	Predictive & Prognostic
HER2	17q12	mutation	10–15%	Similar to EGFR;	Predictive #x0026; Prognostic
DDR2	1q23.3	mutation	4% of LSCCs	a RTK that binds collagen and involves in the regulation of cell growth, differentiation, and metabolism.	Predictive
ALK	2p23.2-p23.1	Chromosomal rearrangement	3–7%	Activate RAS-MEK-ERK, JAK-STAT3, and PI3K-AKT pathways central to proliferation and growth through	Predictive & Not prognostic
ROS1	6q22.1	Chromosomal rearrangement	1–2%	involve in the signal passway including JAK-ATAT3N, RAS/MEK/ERK, PI3K/AKT, and so on, to regulate cellular survival and proliferation in NSCLCs	Predictive & Not prognostic
RET	10q11.21	chromosomal rearrangement	1%	similar to ROS1	Not Predictive & Prognostic
MET	7q31.2	amplification	2–4%	associated with multiple human cancers	Prognostic (Negatively) only
FGFR1	8p11.23	amplification	20%	leads to downstream signaling via PI3K-Akt and RAS-MEK-MAPK related to the growth and survival of tumors	Prognostic (Negatively) only
PDGFRA	4q12	amplification	little	Influence tumor progression	Not Predictive & Not Prognostic
KRAS	12p12.1	mutaion	∼25% of adenocarcinomas	Involve in the RAS/RAF/ERK signal pathway to reguate cell growth, differentiation, and metabolism and promote the survial of tumors	Predictive & Prognostic
BRAF	7q34	mutation	1–3%	Simiar to KRAS	Not Predictive & Prognostic
PIK3CA	3q26.32	mutations	2%	heighten lipid kinase activity and constitutive PI3K-AKT signaling	Prognostic
PTEN	10q23.31	mutation	little	encode a lipid phosphatase that negatively regulates the PI3K-AKT pathway	Predictive & Prognostic
H3F3A	1q42.12	mutation	little	associated with lung cancer progression and promotes lung cancer cell migration by activating metastasis-related genes.	prognostic

### Challenges

Over the years, the knowledge about NSCLCs have been increasing not only in numbers but also in depth. Nonetheless, there exist some disputes and emerging questions on characteristics, detection methods, and targeted therapies about the driver genes in NSCLC.

First, gold standard simplifying problems is our pursuit all the time for everything, of course, including the research about driver genes in NSCLC. One typical example is the algorithms to screen driver genes. It is true that multifarious algorithms effectively improve the discovery about driver genes but different algorithms have different conclusions. It is difficult to determine which one is the most authoritative conclusion. In other words, there is no gold standard for algorithms. Thus, a rounded system to evaluate these prediction methods and analysis these results are urgent. Some databases such as Driver DB (an exome sequencing database for cancer driver genes identification) and Candidate Cancer Gene Database (a database of cancer driver gene from forward genetic screens in mice) have broken the ice and brought dawn. Simultaneously, we need to optimize these existing algorithms and seek a perfect algorithm by experimental verification. Besides, the experimental methods have the same questions as algorithms. FISH, DISH (dual *in situ* hybridization), RT-PCR, IHC, and NGS have their own merits and demerits and how to choose the methods should depend on molecular profiles of the detected driver gene as described above.

Second, we know that the list of driver genes has been adding, which represents the more comprehensive awareness of NSCLC. The signaling pathways in NSCLC make us more clear the relationships within driver genes and the machines of tumorigenesis. Meanwhile, we can base on the signaling pathways to find driver genes. So more targets could be found and applied to clinical tests and targeted therapies [86–90]. As for the characteristics about new driver genes in NSCLC, learning from the analogous well-study driver genes in NSCLC or other cancers is an excellent method.

Last, targeted therapies against driver genes are the optimal choice for advanced patients. To date, some molecular-targeted drugs have applied to clinical trials and evaluated the efficacy. However, the same drugs against driver genes may have different efficacy for different patients because of heterogeneity in different patients. It is necessary to develop personalized therapy. The personalized therapy depending on personal characters can provide patients in NSCLC with the special strategy, which has higher cure rate and reduces the side effects.

In brief, the progress in the way to overcome NSCLC have been gotten but more efforts and energies still need to be injected.
